# Post‐Fabrication Phase Correction of SOI 4 × 4 MMI‐Based 90° Optical Hybrids via Femtosecond‐Laser Trimming

**DOI:** 10.1002/nap2.70320

**Published:** 2026-10-18

**Authors:** Jia Du, Xiongping Bao, Jintian Lin, Xiao Chen, Wenbing Jiang, Boyu Zhang, Yiti Xiong, Wenjun Li, Libing Zhou

**Affiliations:** ^1^ Wangzhijiang Innovation Center for Laser, Aerospace Laser Technology and System Department Shanghai Institute of Optics and Fine Mechanics Chinese Academy of Sciences Shanghai China; ^2^ Center of Materials Science and Optoelectronics Engineering University of Chinese Academy of Sciences Beijing China; ^3^ State Key Laboratory of Ultra‐intense Laser Science and Technology Shanghai Institute of Optics and Fine Mechanics Chinese Academy of Sciences Shanghai China

**Keywords:** 90°optical hybrid, femtosecond‐laser trimming, multimode interference, post‐fabrication correction, silicon photonics

## Abstract

Integrated 90° optical hybrids are essential passive components in coherent receivers, yet their quadrature accuracy and power balance are highly susceptible to fabrication‐induced deviations. Here, we demonstrate postfabrication phase trimming of silicon‐on‐insulator 4 × 4 multimode‐interference‐based 90° optical hybrids using localized femtosecond‐laser modification. The hybrid is implemented with a shallow‐etched rib multimode section and characterized by mapping the relative output phase onto wavelength‐dependent interference spectra. As‐fabricated devices show phase deviations exceeding the ± 5° target and exhibit device‐to‐device performance fluctuations among nominally identical structures. Raman spectroscopy and high‐resolution transmission electron microscopy confirm that femtosecond‐laser irradiation induces a localized crystalline‐to‐amorphous transformation in silicon, introducing a permanent refractive‐index perturbation. By selectively modifying the MMI region, the modal phase evolution and output phase balance can be adjusted after fabrication. After trimming, the phase deviations are reduced to within ± 5° across the full C‐band, accompanied by recovery of the quadrature response, while maintaining common‐mode rejection ratio above 20 dB. These results demonstrate a permanent and power‐free calibration route for coherent silicon photonic circuits.

## Graphical abstract

Fabrication‐induced phase deviations in integrated silicon 4 × 4 MMI‐based 90° optical hybrids are compensated through localized femtosecond‐laser trimming. Permanent refractive‐index modification enables device‐specific postfabrication phase control, reducing phase deviations to within ± 5° across the full C‐band (1530–1565 nm) while maintaining a common‐mode rejection ratio above 20 dB, thereby providing a practical pathway toward high‐precision integrated silicon‐photonic coherent receivers.

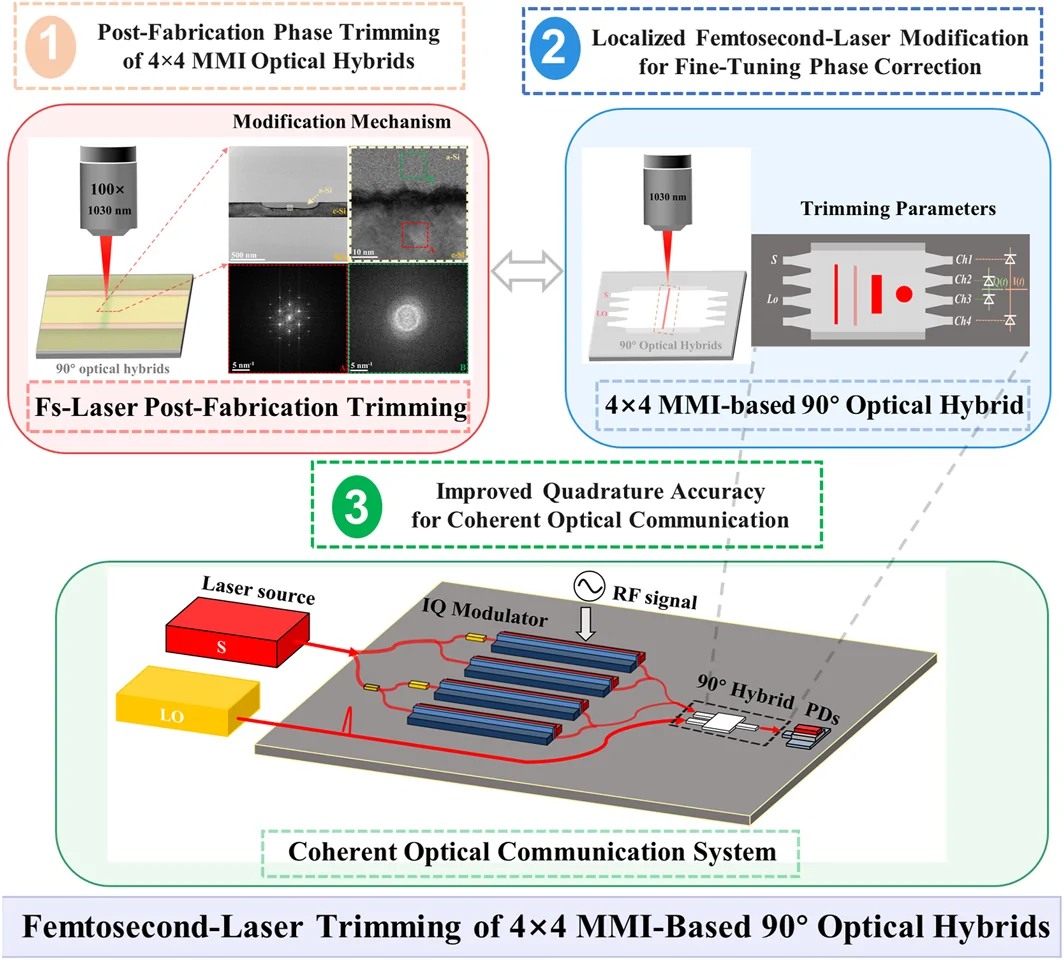

## 1 Introduction

Coherent optical communication has become a central technology for high‐capacity optical links because it enables simultaneous recovery of optical amplitude and phase and supports advanced modulation formats with high spectral efficiency [1, 2, 3]. In integrated coherent receivers, the 90° optical hybrid serves as the passive front‐end component that mixes the received signal with the local oscillator and generates four outputs with prescribed relative phase relationships [4, 5, 6, 7, 8, 9, 10]. The accuracy of this hybrid directly affects balanced photodetection, carrier recovery, and digital signal processing. Therefore, precise quadrature phase relations, low excess loss, and balanced output power are essential for practical coherent photonic integrated circuits. Several integrated 90° optical‐hybrid architectures have been reported, including cascaded 3‐dB directional couplers, multimode‐interference (MMI) couplers, and polarization‐assisted configurations [7, 11, 12, 13, 14]. Among these schemes, 4 × 4 MMI‐based optical hybrids are particularly attractive because they offer compact footprints, passive quadrature operation, and relatively relaxed fabrication requirements. The silicon‐on‐insulator (SOI) platform further provides strong optical confinement, low propagation loss, and compatibility with mature CMOS‐compatible processes. These advantages make SOI‐based 4 × 4 MMI hybrids promising candidates for compact coherent receivers and large‐scale silicon photonic integration.

Nevertheless, the nominal fabrication tolerance of MMI couplers does not necessarily guarantee high‐performance coherent detection. In practical coherent receivers, the phase deviation between the in‐phase (I) and quadrature (*Q*) outputs is commonly required to remain within ± 5°, which is widely adopted as a design target for 90° optical hybrids [15, 16, 17]. Previous studies have shown that fabricated MMI‐based optical hybrids can exhibit phase errors larger than this tolerance even with optimized designs [18, 19, 20]. Small variations in MMI width, etch depth, sidewall profile, and access‐waveguide placement can modify the effective modal indices and accumulated propagation phases in the multimode region. Consequently, fabricated devices may show both phase imbalance and output‐power mismatch, which degrade the common‐mode rejection performance of balanced detection and ultimately limit receiver performance. Post‐fabrication trimming provides an effective route to compensate such deviations after wafer processing. Thermo‐optic and carrier‐based electro‐optic tuning can correct phase errors, but they require continuous electrical power and additional routing complexity [21, 22, 23, 24]. In contrast, femtosecond‐laser processing can introduce permanent and localized material modification with high spatial resolution and negligible static power consumption. In silicon photonics, femtosecond‐laser irradiation can locally transform crystalline silicon into amorphous silicon (a‐Si), thereby changing the refractive index and optical path length. Although this mechanism has been widely explored for trimming single‐mode waveguides and resonant devices [25, 26, 27, 28, 29, 30], its application to multimode‐interference optical hybrids, where multiple modes determine the output phase relationship, remains largely unexplored.

In this work, we demonstrate femtosecond‐laser postfabrication phase trimming of SOI‐based 4 × 4 MMI 90° optical hybrids. We first optimize the MMI geometry and analyze its sensitivity to fabrication‐induced deviations. Fabricated devices are then characterized using a 1 × 2 MMI splitter and a Mach–Zehnder delay interferometer (MZI), which converts the relative output phase errors into wavelength‐dependent interference spectra. Raman spectroscopy and high‐resolution transmission electron microscopy (HRTEM) confirm localized silicon amorphization induced by femtosecond‐laser irradiation. By exploiting this controllable refractive‐index perturbation, we locally modify the MMI region to engineer modal phase accumulation and recover the quadrature response. After trimming, the hybrids show phase deviations within ± 5° across the full C‐band and maintain common‐mode rejection ratios (CMRRs) above 20 dB.

## 2 Device Design and Experimental Methods

### 2.1 Operating Principle of the 4 × 4 MMI‐Based 90° Optical Hybrid

Figure 1 illustrates the operating principle of the 4 × 4 MMI‐based 90° optical hybrid in a coherent receiver. The signal(S) and local oscillator (LO) fields are injected into two input ports of the multimode section, where multiple lateral modes are excited and interfere during propagation. According to the self‐imaging principle [31], the beat length of the two lowest‐order modes can be written as follows:

(1) $$L_{\pi }=\frac{\pi }{\beta_{0}-\beta_{1}}\approx \frac{4n_{r}W_{e}^{2}}{3\lambda },$$

where *β*_0_ and *β*_1_ are the propagation constants of the fundamental and first‐order modes, respectively, *W*_*e*_ is the effective width of the MMI region, *n*_*r*_ is the effective refractive index of the rib multimode waveguide, and *λ* is the free‐space wavelength. By properly optimizing W_MMI_ and L_MMI_, the four output ports can achieve nearly equal power distribution and a prescribed relative phase relationship. For a 4 × 4 MMI coupler, the relative output phase *φ*_*x,y*_ between input port *x* and output port *y*, after removing the common phase terms, follows the standard MMI phase relation [32, 33]:

(2) $$\phi_{x,y}=\frac{15}{32}\pi -\frac{\pi }{2}{\left(-1\right)}^{x+y}+\frac{\pi }{16}\times \left[x+y-x^{2}-y^{2}+{\left(-1\right)}^{x+y}\left(2xy-x-y+\frac{1}{2}\right)\right]$$

**FIGURE 1:**
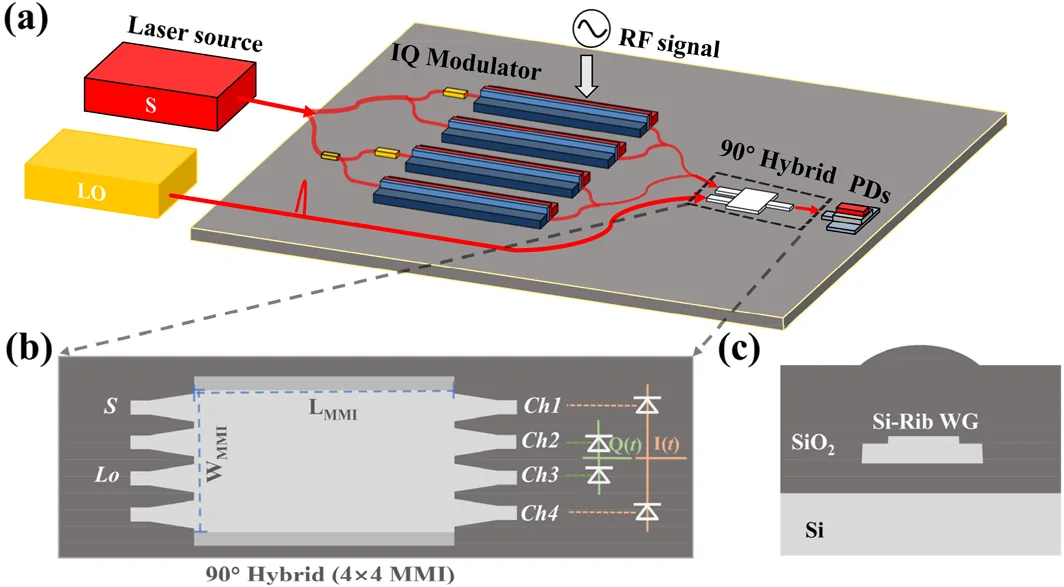
Concept and operating principle of the 4 × 4 MMI‐based 90° optical hybrid for coherent detection. (a) Schematic of a coherent receiver including the S input, LO input, 90° optical hybrid, and balanced photodetectors (PDs). (b) Enlarged view of the SOI 4 × 4 MMI hybrid, where the S and LO are injected into two input ports and four phase‐shifted outputs are generated. (c) Cross‐sectional schematic of the SOI‐based rib‐waveguide 4 × 4 MMI hybrid.

In this work, channel 1(Ch1) is selected as the phase reference. The ideal relative output phase sequence is defined as 0°, 270°, 90°, and 180° for Ch1–Ch4, respectively. The phase deviations are defined as the wrapped difference between the measured relative phase and the ideal phases, for example Δ*φ*_21_ = (*φ*_2_ − *φ*_1_ − 270°).

### 2.2 Design Optimization and Fabrication‐Tolerance Analysis

The 90° optical hybrid was designed on a 220‐nm‐thick SOI platform using a shallow‐etched rib waveguide as schematically shown in Figure 1b,c. Linear tapers were inserted between the single‐mode access waveguides and the multimode region to reduce mode mismatch and coupling loss. The MMI geometry was optimized by sweeping L_MMI_ from 101 to 113 μm and W_MMI_ from 7.25 to 7.55 μm using eigenmode and propagation simulations. Figure 2 summarizes the phase‐deviation evolution under different geometrical parameters. The phase response exhibits stronger sensitivity to W_MMI_ than to L_MMI_, which is expected because the MMI width directly determines the effective width *W*_*e*_, modal propagation constants, and accumulated phase differences among supported modes. The optimized geometry uses *L*_MMI_ ≈ 105.5 μm. For this geometry, the simulated phase deviation remains within ± 5° over the target wavelength range, and the simulated excess loss remains below 0.5 dB, reaching approximately 0.03 dB near 1550 nm.

**FIGURE 2:**
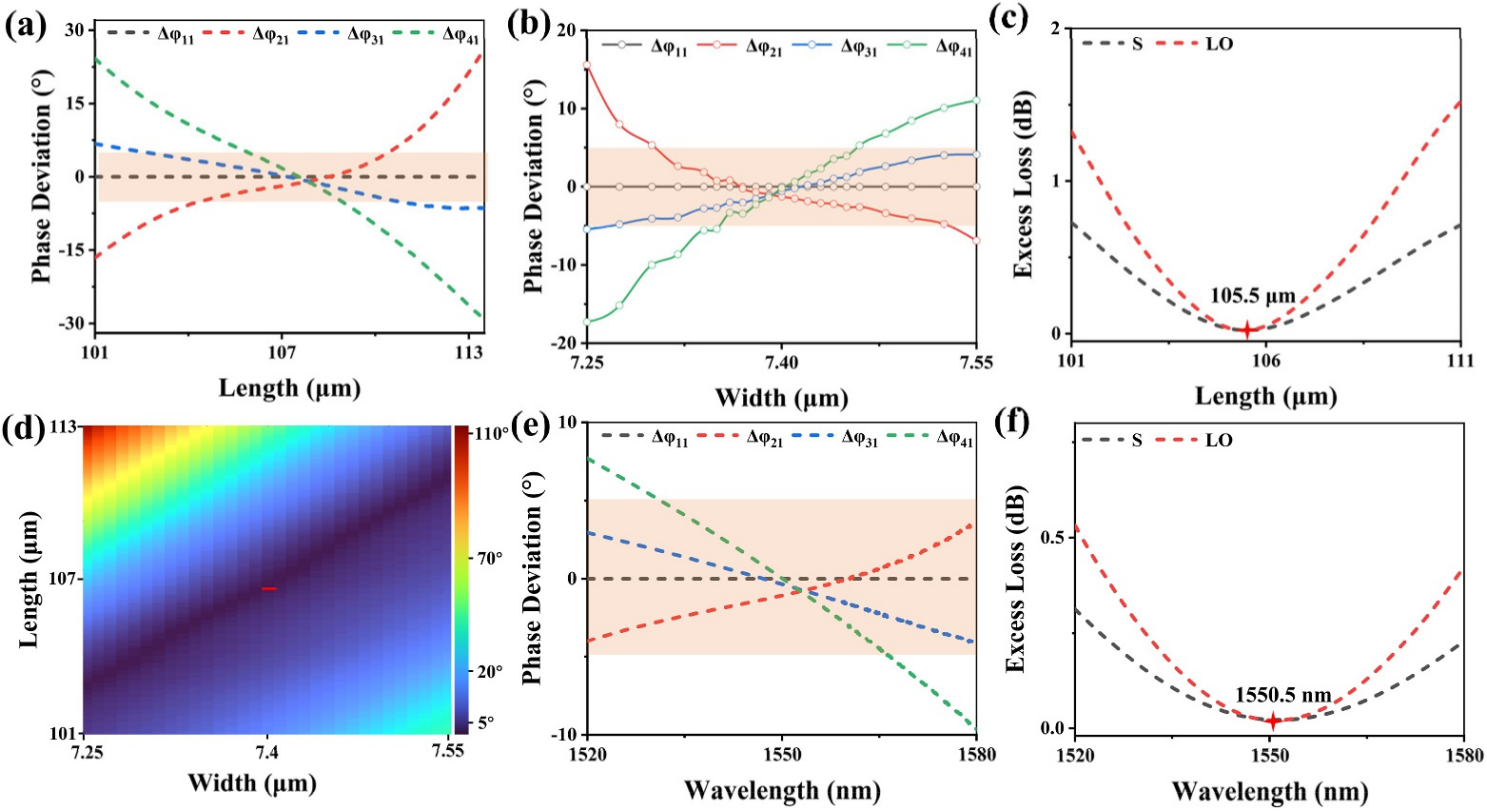
Design optimization and fabrication‐tolerance analysis of the 4 × 4 MMI optical hybrid. (a) Phase‐deviation evolution as a function of MMI length. (b) Phase‐deviation evolution as a function of MMI width. (c) Excess loss as a function of MMI length. (d) Two‐dimensional tolerance map of phase deviation under simultaneous variations of L_MMI_ and W_MMI_. The red box indicates the assumed fabrication‐error window. (e) Simulated wavelength‐dependent phase deviation of the optimized structure. (f) Simulated wavelength‐dependent broadband excess loss.

A two‐dimensional tolerance map was further calculated by simultaneously varying L_MMI_ and W_MMI_. When dimensional deviations within a representative ± 5 nm fabrication window are considered, some output channels exceed the ± 5° phase‐deviation tolerance. This result indicates that although the MMI exhibits relatively robust power splitting characteristics, its phase response remains sensitive to fabrication‐induced dimensional variations. Therefore, devices with *L*_MMI_ = 103, 105, and 107 μm were fabricated on the same platform to introduce representative phase deviations and evaluate the effectiveness of the proposed postfabrication trimming approach.

### 2.3 Sample Preparation and Material Characterization

The test structure consists of a 1 × 2 MMI splitter, a MZI delay interferometer, and the 4 × 4 MMI optical hybrid (Figure 3a). The delay interferometer introduces a wavelength‐dependent phase difference between the two inputs of the optical hybrid, enabling extraction of the relative quadrature phase response from measured transmission spectra. The optical hybrid was designed and optimized for TE‐mode operation. To this end, a TE polarizer was inserted in the optical path to fix the input polarization throughout all measurements before and after trimming, and the device was positioned on a micromechanical coupling platform equipped with a temperature‐controlled stage to minimize temperature‐induced variations during measurement. Since the input polarization state was not systematically varied, polarization‐dependent loss (PDL) was not evaluated and the results pertain specifically to TE‐polarized operation. Polarization‐dependent effects induced by localized amorphization merit further investigation and will be addressed in our future studies.

**FIGURE 3:**
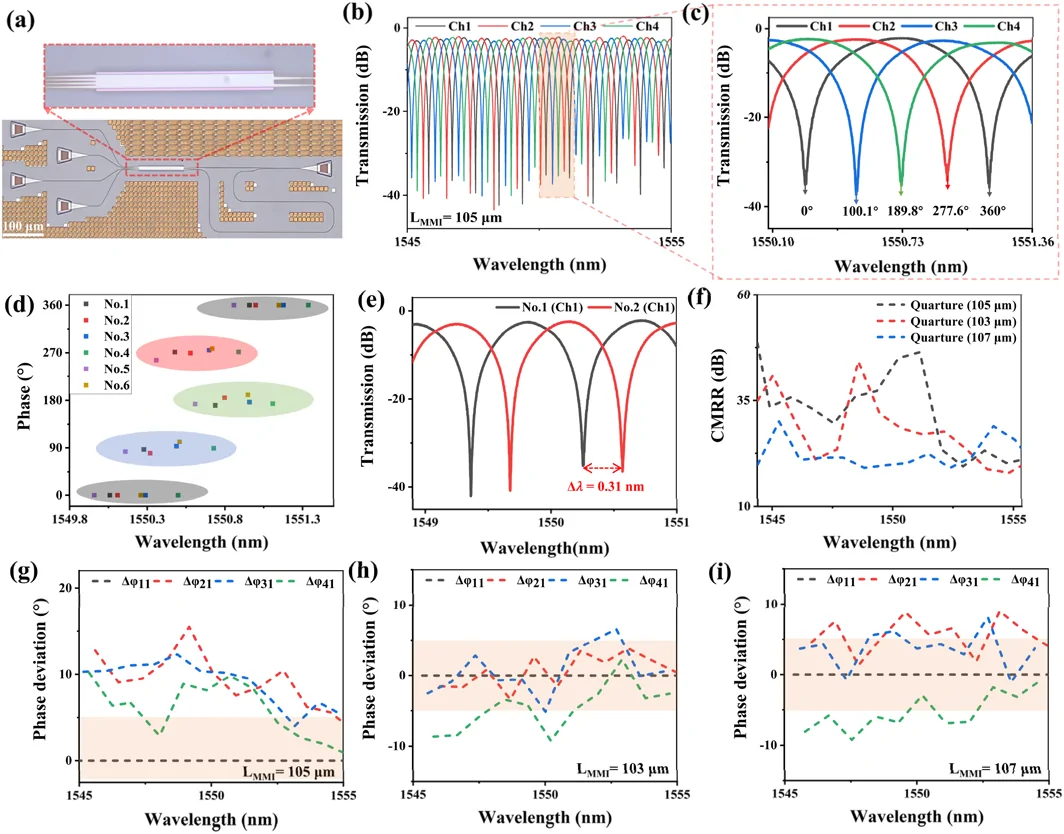
As‐fabricated performance of the 90° optical hybrid. (a) Optical microscope image of the fabricated 4 × 4 MMI‐based test structure. (b) Measured transmission spectra of the four output channels around 1550 nm at *L*_MMI_ = 105 μm. (c) Representative one‐FSR spectral window used for phase extraction. (d) Device‐to‐device variation among six nominally identical structures at *L*_MMI_ = 105 μm. (e) Ch1 spectral deviation between two nominally identical 105‐μm devices. (f) CMRR variation for devices with different MMI lengths. (g–i) Wavelength‐dependent phase deviations extracted from the measured spectra for *L*_MMI_ = 105, 103, and 107 μm, respectively.

Postfabrication trimming was performed by focusing 1030‐nm femtosecond laser pulses onto the device through a 100X objective. The laser source (PHAROS, Light Conversion) provided pulses with a duration of approximately 290 fs and a repetition rate of 250 kHz, producing a focal spot of approximately 1 μm in diameter for localized modification. The single‐pulse fluence was varied from 0.52 to 0.92 J/cm^2^ to investigate the influence of laser energy on the trimming performance. Before each experiment, the sample was carefully leveled, and an autofocus algorithm was applied to maintain a stable focal position. The samples were scanned using an Aerotech air‐bearing linear translation stage at a speed of 30 mm/s, and the laser pulse energy was calibrated before each experiment. The laser‐induced material evolution was characterized using HRTEM and Raman spectroscopy to elucidate the physical mechanism of femtosecond‐laser‐induced trimming.

## 3 Results and Discussion

### 3.1 As‐Fabricated Device Performance

Figure 4 shows the simulated operation of the optimized 4 × 4 MMI optical hybrid under TE polarization at *λ* = 1550 nm. When the S and LO are injected into the designated input ports, the multimode interference pattern evolves along the MMI region and generates four outputs with the designed relative phase relationship. As the input phase difference is varied, the four output powers exhibit sinusoidal oscillations. The extracted phase positions correspond to the designed quadrature relationship, confirming that the optimized geometry supports coherent I/*Q* mixing.

**FIGURE 4:**
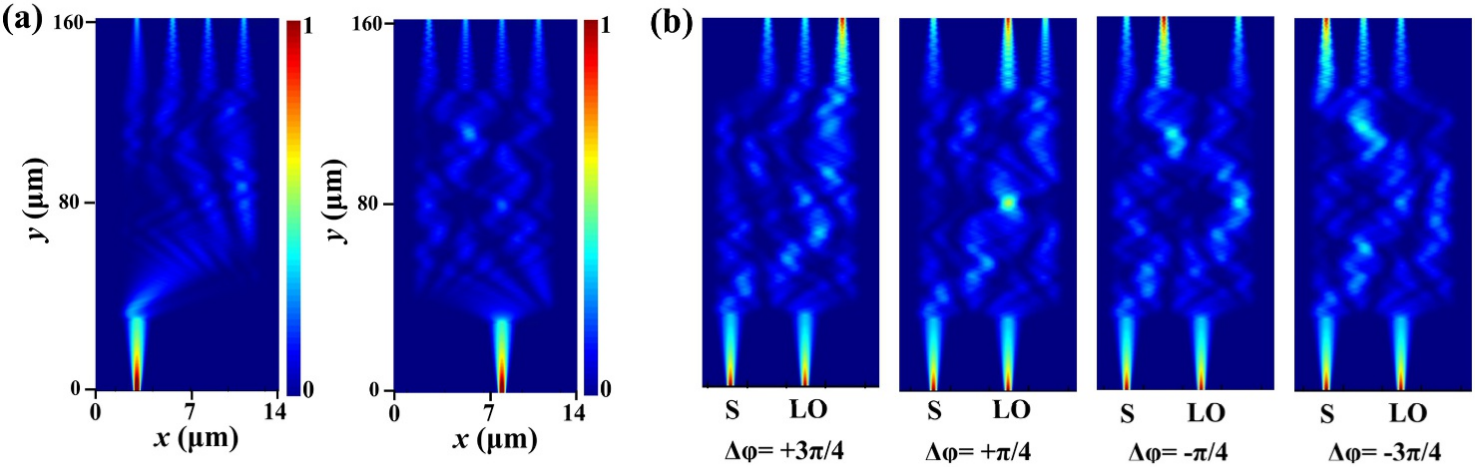
Simulated optical‐field evolution of the optimized 4 × 4 MMI optical hybrid at 1550 nm. (a) Optical‐field distributions for separate S and LO excitation in the MMI region. (b) Optical‐field distributions for simultaneous excitation with different initial phase differences.

The fabricated 90° optical hybrids were subsequently characterized to evaluate their post‐fabrication performance (Figure 3). The four output spectra around 1550 nm show periodic interference fringes with an FSR of approximately 0.9 nm. By comparing the relative spectral positions among the four channels, the phase deviations of Ch2–Ch4 with respect to Ch1 were extracted. Several channels exhibit phase deviations beyond the ± 5° tolerance, indicating that fabrication‐induced variations perturb the designed quadrature condition (Figure 3g). Device‐to‐device comparison further confirms the phase sensitivity of the MMI structure. Six nominally identical devices with *L*_MMI_ = 105 μm exhibit noticeable variation in the extracted phase deviations near the target wavelength (Figure 3d). Figure 3e compares the Ch1 spectra of two nominally identical devices and shows a spectral shift of approximately 0.31 nm, reflecting fabrication‐induced variations in the optical path length and phase response. Additional devices with *L*_MMI_ = 103 and 107 μm were also characterized. The 103‐μm devices show relatively smaller phase deviations, whereas the 107‐μm devices exhibit larger phase imbalance. All fabricated devices maintain CMRRs near 20 dB for the *Q* differential pairs (Figure 3f), indicating acceptable power balance but insufficient phase accuracy. Therefore, the subsequent femtosecond‐laser trimming experiments focus on the 105‐μm optical hybrids.

### 3.2 Femtosecond‐Laser‐Induced Modification

To verify the material modification induced by femtosecond‐laser irradiation, TEM characterizations were performed on laser‐processed silicon regions as shown in Figure 5. Cross‐sectional HRTEM images provide direct structural evidence of the localized amorphous region. The modified silicon region is spatially confined, with a clear interface between the amorphous layer and the underlying crystalline silicon. Fast Fourier transforms (FFT) patterns extracted from the HRTEM image further distinguish the crystalline and amorphous regions.

**FIGURE 5:**
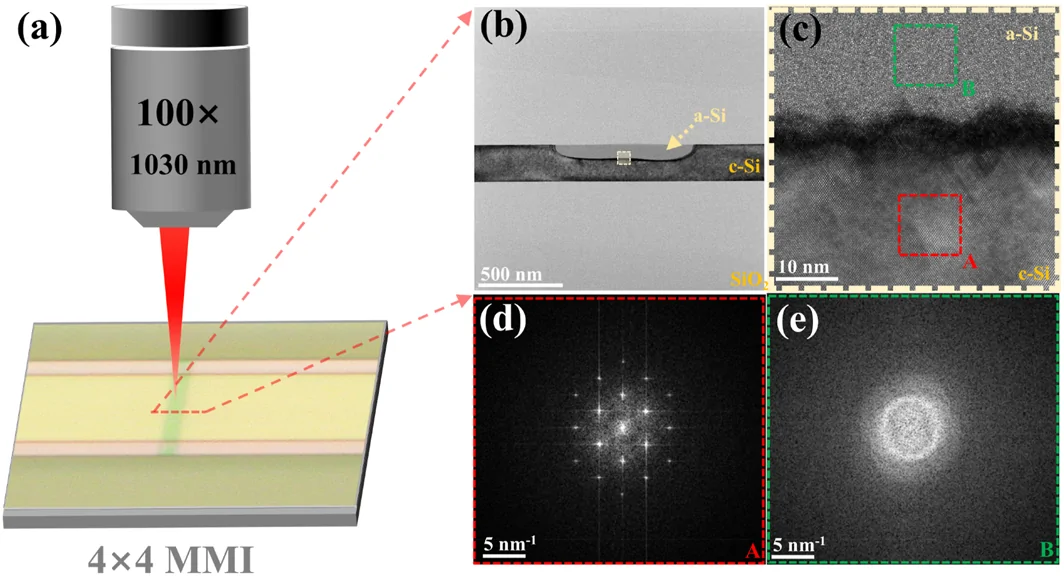
TEM characterization of femtosecond‐laser‐modified silicon. (a) Schematic of the femtosecond‐laser modification setup. (b) Cross‐sectional TEM image of the modified region. (c) Enlarged HRTEM image, where region A corresponds to crystalline silicon and region B corresponds to laser‐induced amorphous silicon. (d, e) FFT patterns extracted from the crystalline and amorphous regions, respectively.

Raman spectroscopy further verifies the laser‐induced amorphization (Figure 6b). The unmodified silicon waveguide exhibits a sharp crystalline silicon Raman peak near 520 cm^−1^. After femtosecond‐laser irradiation, this peak is significantly suppressed, accompanied by the emergence of a broad band near 480 cm^−1^, indicating the formation of amorphous silicon. Furthermore, Raman mapping at 480 cm^−1^ confirms the localized and controllable distribution of the laser‐modified amorphous region (Figure 6a). The dependence of amorphization behavior on laser fluence and the corresponding Raman evolution under different processing conditions are further investigated in Supporting Information S1: Figure S1.

**FIGURE 6:**
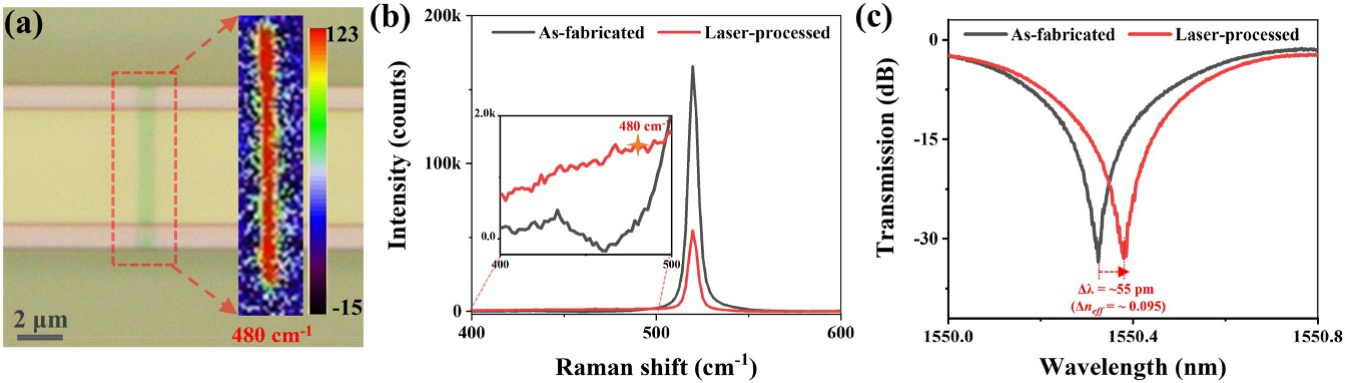
Raman characterization and optical calibration of femtosecond‐laser‐induced modification. (a) Schematic of the femtosecond‐laser modification region and Raman mapping at 480 cm^−1^. (b) Raman spectra of the silicon waveguide region before and after laser irradiation. (c) Calibration of laser‐induced effective‐index change using a single‐mode MZI test structure.

To quantify the optical impact of the laser‐induced material modification, single‐mode MZI structures were employed for calibration. After laser irradiation, the interference spectra exhibit a red shift, indicating an increased effective optical path length. Under the investigated processing conditions, the measured resonance shift is approximately 0.055 nm, whereas the additional insertion loss is approximately 0.2 dB. Considering the effective laser‐modified region identified by material characterization, the corresponding effective‐index change (Δn_*eff*_) extracted from the measured wavelength shift is estimated to be approximately 0.095. Here, Δn_*eff*_ represents the mode‐weighted effective‐index variation induced by the localized amorphous region. The relationship between the modified a‐Si region, optical mode overlap, and the resulting effective‐index perturbation is further analyzed in Supporting Information S1: Figure S2, where the corresponding material‐level refractive‐index perturbation is quantitatively estimated. This analysis demonstrates that femtosecond‐laser‐induced amorphization introduces sufficient optical phase perturbation for postfabrication trimming.

### 3.3 Post‐Fabrication Trimming of 4 × 4 MMI‐Based 90° Optical Hybrids

Based on the confirmed material‐modification mechanism, targeted femtosecond‐laser trimming was applied to MMI‐based 90° optical hybrids. The localized amorphous‐silicon modification can effectively modify the output phase relationship of the 4 × 4 MMI optical hybrid. This effect originates from the laser‐induced refractive‐index perturbation, which modifies the propagation constants of different modes and introduces mode‐dependent phase accumulation within the multimode region. As a result, the modal interference and self‐imaging condition can be locally adjusted whilst maintaining the overall MMI operation. The influences of modification position, modified‐region dimensions, and refractive‐index perturbation on the phase evolution and trimming capability are further investigated through numerical simulations in Supporting Information S1: Figure S2. Considering the complex modal interactions and device‐to‐device variations of 4 × 4 MMI‐based 90° optical hybrids, device‐specific trimming configurations are adopted to compensate for fabrication‐induced phase deviations.

In the postfabrication trimming experiments, the laser‐modified region was positioned near the longitudinal center of the MMI section, and two representative modification geometries were investigated: a full‐width transverse line and a localized line as illustrated in Figure 7a–d, respectively. After femtosecond‐laser trimming, the transmission spectra of the four output channels exhibit slight red shifts, corresponding to an increased effective optical path length induced by local amorphization. For full‐width transverse‐line modification, the induced refractive‐index perturbation interacts relatively uniformly with the multimode region, resulting in similar spectral shifts among the four output ports (Figure 7b). The total additional insertion loss of the hybrid remains within approximately 2.2 dB around 1550 nm, mainly originating from laser‐induced material modification and associated scattering. The additional loss consumes part of the coherent receiver's optical power budget, with its impact on sensitivity depending on the laser‐trimmed location and receiver configuration. Beam‐shaping techniques, such as flat‐top illumination, combined with refined processing parameters, are expected to improve modification uniformity and thereby reduce the trimming‐induced loss. Detailed analyses of the loss characteristics and its system‐level effect, together with possible optimization strategies, are provided in Supporting Information S1: Figure S3.

**FIGURE 7:**
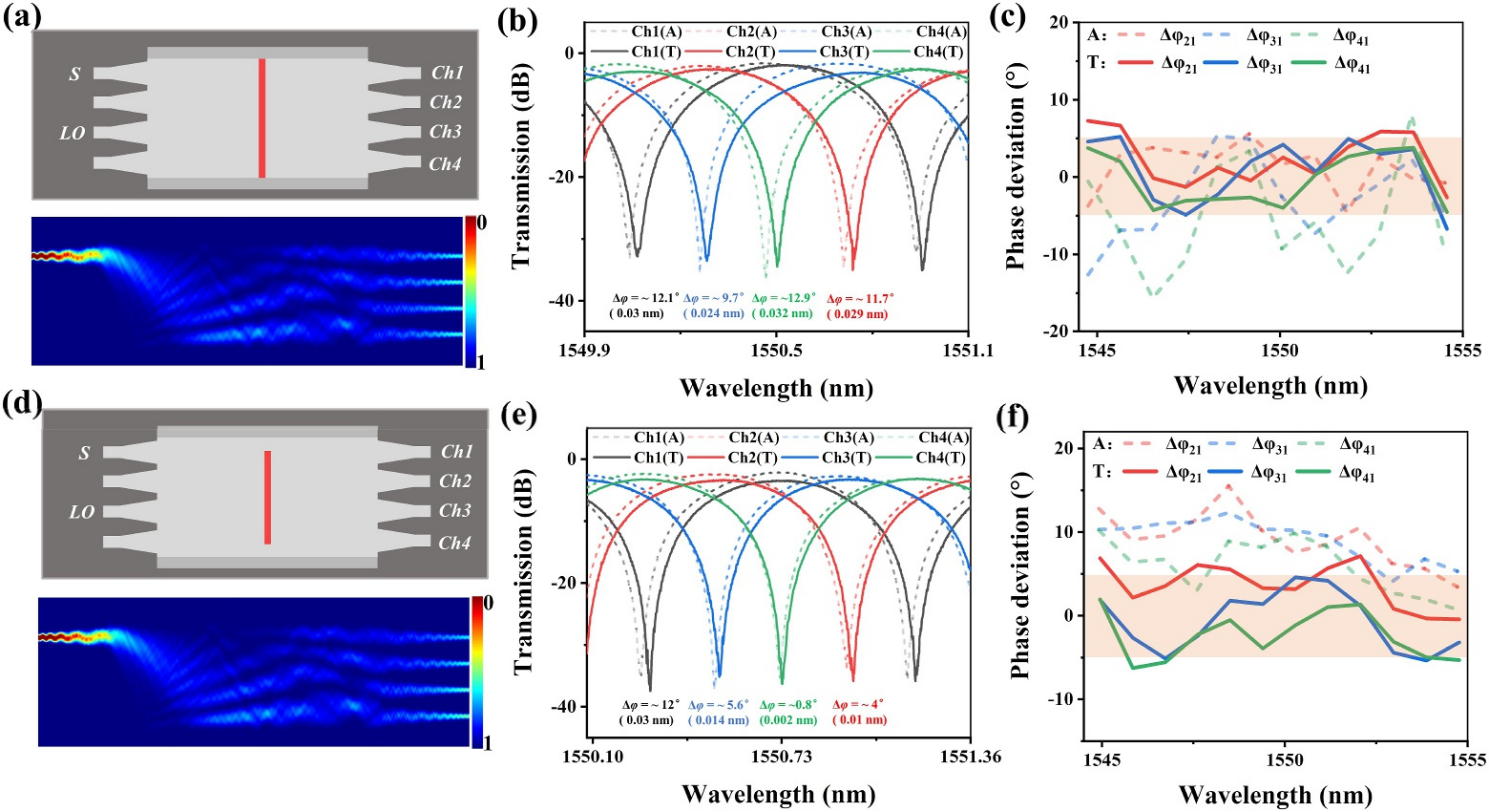
Postfabrication phase trimming of the 4 × 4 MMI‐based optical hybrid using localized femtosecond‐laser modification. (a–c) Full‐width transverse‐line modification across the 7.4‐μm‐wide central MMI region: (a) schematic of the full‐width transverse‐line modification and simulated optical‐field distributions, (b) measured spectra around one FSR near 1550 nm, and (c) phase‐deviation evolution over 1545–1555 nm. (d–f) Localized‐line modification across the 6.5‐μm‐wide central region: (d) schematic of the localized‐line modification and simulated optical‐field distributions, (e) measured spectra around one FSR near 1550 nm, and (f) phase‐deviation evolution over 1545–1555 nm. The labels A and T denote the as‐fabricated and trimmed states, respectively.

In contrast, the localized‐line modification selectively interacts with the nonuniform multimode field, enabling port‐dependent spectral shifts and differential phase correction (Figure 7e). By exploiting the mode‐dependent phase perturbation introduced by the localized amorphous region, femtosecond‐laser trimming effectively compensates for fabrication‐induced phase deviations and restores the quadrature phase response within the ± 5° tolerance window near 1550 nm, referenced to the Ch1 (Figure 7c–f). The broadband phase response and phase‐deviation evolution across the full C‐band (1530–1565 nm) are further analyzed in Supporting Information S1: Figure S3, demonstrating the effectiveness of the proposed trimming approach over the full C‐band.

The measured phase‐deviation evolution confirms that the quadrature relationship is restored to within ± 5° near 1550 nm after trimming as shown in Figure 8a. Meanwhile, the CMRR remains above 20 dB around the target wavelength (Figure 8b), indicating that the phase correction is achieved whilst maintaining the output‐power balance required for balanced coherent detection. The long‐term stability was further evaluated by remeasuring the trimmed devices after 90 days under identical measurement conditions. The spectra, represented by the Ch1 response in Figure 8c, show negligible change, demonstrating the long‐term stability of the femtosecond‐laser‐induced phase correction. The other three channels exhibited essentially identical behavior with similarly negligible changes, consistent with the intrinsic stability of the laser‐induced a‐Si phase.

**FIGURE 8:**
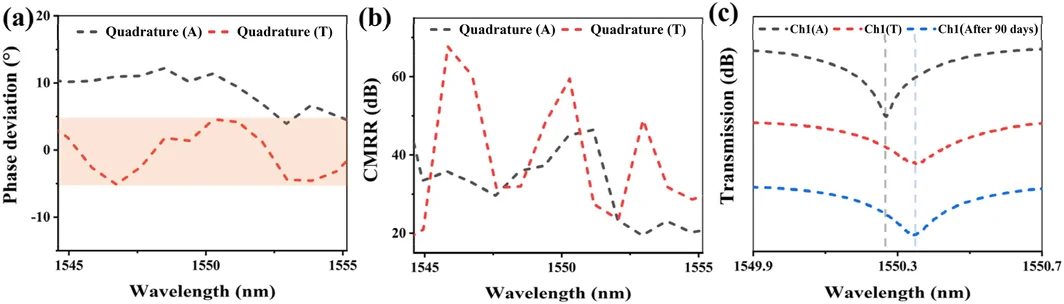
Performance variations of the optical hybrid after femtosecond‐laser trimming. (a) Quadrature phase deviation of Ch3 relative to Ch1, referenced to the ideal 90° quadrature relationship. (b) CMRR variation of the quadrature channel formed by Ch2 and Ch3. (c) Spectra of the trimmed hybrid measured initially and after 90 days on a temperature‐controlled stage to maintain identical measurement conditions, demonstrating the long‐term stability of the femtosecond‐laser‐induced phase correction.

These results experimentally validate the proposed trimming mechanism: femtosecond‐laser‐induced local amorphization modifies the effective refractive index, changes modal phase accumulation in the MMI region, and compensates fabrication‐induced phase imbalance. Unlike thermo‐optic or carrier‐based tuning approaches, this correction is permanent and requires no static electrical power. Therefore, the proposed approach provides a promising route toward wafer‐level calibration and yield enhancement of integrated coherent photonic receivers.

## 4 Conclusion

We have demonstrated femtosecond‐laser postfabrication trimming of SOI‐based 4 × 4 MMI 90° optical hybrids. Although the optimized MMI design provides low excess loss and nominal quadrature operation, fabricated devices exhibit phase deviations beyond the ± 5° tolerance and device‐to‐device variations in phase response due to process‐induced variations in the multimode section. Raman spectroscopy and TEM characterization confirm that femtosecond‐laser irradiation produces localized silicon amorphization, which introduces a permanent refractive‐index perturbation. By selectively modifying the MMI region, the modal phase accumulation can be adjusted after fabrication, enabling compensation of phase imbalance and restoration of the quadrature phase relationship. The trimmed devices achieve phase deviations within ± 5° across the full C‐band whilst maintaining CMRR values above 20 dB. This work provides a permanent postfabrication correction strategy without static power consumption for coherent silicon photonic receivers and offers a practical pathway toward wafer‐level calibration and yield enhancement of high‐performance photonic integrated circuits.

## Author Contributions

**Jia Du:** validation, software, writing – original draft, data curation, methodology, conceptualization. **Xiongping Bao:** methodology, software, investigation, conceptualization, data curation, writing – original draft. **Jintian Lin:** formal analysis, validation, data curation. **Xiao Chen:** investigation, validation. **Wenbing Jiang:** methodology, investigation. **Boyu Zhang:** investigation, formal analysis. **Yiti Xiong:** validation, formal analysis. **Wenjun Li:** visualization, validation. **Libing Zhou:** writing – review and editing, formal analysis, resources, funding acquisition.

## Conflicts of Interest

The authors declare no conflicts of interest.

## Supporting information


Supporting Information S1

## Data Availability

The data that support the findings of this study are available from the corresponding author upon reasonable request.
