# Supplementary material for: Post‐Fabrication Phase Correction of SOI 4 × 4 MMI‐Based 90° Optical Hybrids via Femtosecond‐Laser Trimming
Source: Nanophotonics. 2026 Oct 18;15(20):e70320. doi: 10.1002/nap2.70320 (PMC13678421; doi:10.1002/nap2.70320)
Supplement: Supplementary file 1 [file NAP2-15-e70320-s001.docx]

Post-Fabrication Phase Correction of SOI 4×4 MMI-Based 90° Optical Hybrids via Femtosecond-Laser Trimming

Jia Du,^1^ Xiongping Bao,^1,2, †^ Jintian Lin,^2,3^ Xiao Chen,^1^ Wenbing Jiang, ^1,2^ Boyu Zhang,^1^ Yiti Xiong,^1^ Wenjun Li,^1,2^ and Libing Zhou,^1,2, *^

*^1^Wangzhijiang Innovation Center for Laser, Aerospace Laser Technology and System Department, Shanghai Institute of Optics and Fine Mechanics, Chinese Academy of Sciences, Shanghai 201800, China*

*^2^Center of Materials Science and Optoelectronic**s Engineering, University of Chinese Academy of Sciences, Beijing 100049, China*

*^3^State Key Laboratory of Ultra-intense Laser Science and Technology, Shanghai Institute of Optics and Fine Mechanics, Chinese Academy of Sciences, Shanghai 201800, China*

^†^These authors contributed equally to this work

^4^lbzhou@siom.ac.cn

S1. Femtosecond-laser-induced amorphization and effective-index modulation in silicon waveguides

The femtosecond-laser processing window of silicon photonic structures was investigated using single-crystal silicon waveguides fabricated on a silicon-on-insulator (SOI) platform. Raman spectroscopy and optical spectral measurements were performed to characterize the structural evolution and optical response induced by femtosecond-laser irradiation. A single-mode Mach–Zehnder interferometer (MZI) was employed to correlate the laser fluence with amorphous-silicon (a-Si) formation and the corresponding effective-index modulation. The pulse fluence was varied from 0.52 to 0.92 J/cm², covering a broad processing window from weak laser-induced modification to progressively enhanced amorphization. The Raman response and resonance wavelength shift (Δλ) were recorded under different processing conditions. For each fluence condition, the Δλ was measured three times to evaluate the repeatability of the laser-induced optical modulation. The results show that increasing laser fluence gradually enhances the crystalline-to-amorphous silicon transition and produces controllable effective-index modulation, providing a quantitative reference for the femtosecond-laser trimming of the 4×4 MMI-based optical hybrid.

Fig. S1. Characterization of femtosecond-laser-induced amorphization and effective-index modulation in silicon waveguides. (a) Raman spectra of single-crystal silicon waveguides modified by femtosecond-laser irradiation under different pulse fluences. The inset shows the enlarged a-Si-related Raman band near 480 cm^-1^ and its evolution with increasing pulse fluence. (b) Resonance wavelength shift (Δλ) and extracted effective-index change (Δn*_eff_*) of the MZI waveguide induced by localized femtosecond-laser modification under different pulse fluences.

The laser-induced amorphous region was further characterized by cross-sectional TEM to verify the structural origin of the refractive-index perturbation. As shown in Fig. S2(a), a localized amorphous region with a clear interface between a-Si and crystalline silicon (c-Si) was observed in the 220-nm-thick silicon waveguide modified at a laser fluence of 0.71 J/cm². The a-Si region extends approximately 850 nm along the waveguide propagation direction with a modified depth of approximately 100 nm. To quantitatively evaluate the optical impact of the irregularly distributed amorphous region, the experimentally observed a-Si profile was approximated by an equivalent cross-sectional profile with a comparable modified area (Fig. S2(b)). Based on the equivalent modified-region geometry and the refractive-index difference between a-Si and c-Si at 1550 nm, the corresponding material-level refractive-index perturbation was estimated to be approximately Δn ≈ 0.14.

The effective-index perturbation extracted from the MZI measurement in the main manuscript (Δn*_eff_ =*0.095) represents the mode-weighted effective-index change, while the estimated Δn ≈ 0.14 corresponds to the material-level refractive-index perturbation of the amorphous region. Considering the optical mode overlap with the modified region, the material-level perturbation results in an effective-index change in good agreement with the experimentally extracted value [S1-S2]. Furthermore, previous studies have reported that a-Si exhibits a higher refractive index than c-Si in the near-infrared region, supporting the physical origin and direction of the refractive-index perturbation induced by femtosecond-laser amorphization [S3–S8]. Future work could further verify the material-level refractive-index perturbation by fabricating larger-area modified regions and performing localized optical spectroscopy or ellipsometry measurements.

Fig. S2. (a) Cross-sectional TEM characterization of the femtosecond-laser-induced amorphous region in single-crystal silicon waveguides. (b) Equivalent geometric profile of the amorphized region derived from TEM characterization for estimating the refractive-index perturbation.

**S2. Design and performance analysis of femtosecond-laser-trimmed 4×4 MMI-based 90° optical hybrids**

The phase accuracy of a 4×4 MMI-based optical hybrid is determined by the relative phase evolution of supported modes within the multimode region. Fabrication-induced dimensional variations modify the modal propagation constants and disturb the designed self-imaging condition, resulting in phase imbalance among the output ports. Femtosecond-laser trimming introduces localized refractive-index perturbations through the formation of a-Si, rather than uniformly modifying the entire MMI region. Due to the distinct spatial distributions of different modes, the localized a-Si region induces mode-dependent phase perturbations, enabling controlled adjustment of the relative modal phases and modification of the output quadrature response.

The effects of different trimming parameters, including modification position, modified-region length and width, and refractive-index perturbation, were systematically investigated. The laser fluence was used as a control parameter to modify the degree of amorphization and the resulting refractive-index perturbation. As shown in Figs. S3–S4, Fig. S3(a) presents the schematic of the 4×4 MMI-based optical hybrid with structural dimensions of L_MMI ×_W_MMI_ = 105 × 7.4 μm. The trimming performance was evaluated under different modification positions (Fig. S3(c)), modified-region lengths at the central MMI region (Fig. S3(d)), modified-region widths at the central MMI region (Fig. S3(e)), and different refractive-index perturbations induced by varying laser fluence (Fig. S3(f)).

Fig. S3. Design schemes for investigating the influence of femtosecond-laser trimming parameters on the phase-control capability of the 4×4 MMI-based 90° optical hybrid. (a) Schematic of the 4×4 MMI-based 90° optical hybrid. (b) Schematic of the femtosecond-laser-modified region. (c) Trimming schemes with different modification positions. (d) Trimming schemes with different modified-region lengths at the central MMI region. (e) Trimming schemes with different modified-region widths at the central MMI region. (f) Trimming schemes with different refractive-index perturbations induced by different laser fluences.

Figure S4 presents the simulated optical-field evolution of the 4×4 MMI-based optical hybrid under representative trimming conditions. The simulation was performed using an MMI section with dimensions of L_MMI ×_W_MMI_ = 105 × 7.4 μm to investigate the phase-control mechanism induced by localized refractive-index perturbations. Figure S4(a) shows the optical-field distribution of the unmodified crystalline-silicon MMI under simultaneous excitation of the two input ports with an initial phase difference of +3π/4, serving as the reference condition.

The central MMI region was selected as the representative trimming location because the optical field undergoes significant evolution during the propagation of the coherent S and LO inputs toward the four output ports. The central region could exhibit stronger interaction with the propagating multimode field, where localized refractive-index perturbations can potentially provide more effective phase modulation.

The simulated results indicate that localized amorphous-silicon modification perturbs the propagation constants of supported modes and alters their relative phase accumulation within the multimode region. Compared with uniform modification, localized trimming introduces stronger mode-selective perturbations due to the different spatial overlap between the modified region and individual modes, enabling flexible adjustment of the relative phases among the four output ports. These results further clarify the physical mechanism of femtosecond-laser-induced post-fabrication phase correction.

Fig. S4. Simulated optical-field evolution of the 4×4 MMI-based optical hybrid at 1550 nm under different trimming conditions. (a) Optical-field distribution of the unmodified crystalline-silicon MMI under simultaneous excitation of the two input ports with an initial phase difference of +3π/4. (b–f) Optical-field evolution under representative trimming conditions, including (b) a central modified-region length of 7.4 μm; (c) a central modified-region length of 7.4 μm with a modified-region refractive index of n=3.7; (d) a central modified-region width of 4 μm; (e) a 7.4-μm-long modified region positioned 10 μm from the input end of the 105-μm-long MMI section; and (f) a central modified-region length of 7.4 μm with a width of 10 μm.

**S3. Broadband performance and optimization of femtosecond-laser-trimmed 4×4 MMI-based 90° optical hybrids**

Figure S5 summarizes the broadband phase-correction performance of the 4×4 MMI-based optical hybrid before and after femtosecond-laser trimming over the C-band (1530–1565 nm). Considering the requirements of 90° optical hybrids in coherent optical communication systems, the phase deviations of the four output ports were analyzed across the entire C-band. Since the free spectral range (FSR) of the device is approximately 0.9 nm, the phase response within a single FSR is insufficient to fully evaluate the broadband phase-correction capability. The results show that femtosecond-laser trimming effectively reduces the phase deviations compared with the as-fabricated devices, with the residual phase deviations maintained mostly within ±5° across the full C-band, referenced to the Channel 1. These results further demonstrate the broadband phase-correction capability of the proposed femtosecond-laser trimming approach.

Fig. S5. Broadband phase correction of the 4×4 MMI-based optical hybrid after femtosecond-laser trimming. Full-width transverse-line modification across the 7.4-μm-wide central MMI region: (a) measured transmission spectra across the C-band and (b) phase-deviation evolution across the C-band. Localized-line modification across the 6.5-μm-wide central region: (c) measured transmission spectra across the C-band and (d) phase-deviation evolution across the C-band. The labels A and T denote the as-fabricated and trimmed states, respectively. The ±5° tolerance band is marked in (b) and (d).

After femtosecond-laser trimming, the additional insertion loss of the 90° optical hybrid was evaluated. The additional insertion loss varies from approximately 0.4 to 0.8 dB among the four output channels, while the total additional insertion loss remains within approximately 2.2 dB around 1550 nm. Based on the modified length of the multimode region, the equivalent loss density introduced by femtosecond-laser modification is roughly estimated to be approximately 0.3 dB/μm, which is comparable to the loss level observed in the single-mode MZI calibration structure.

Regarding the system-level impact, the additional loss reduces the optical power available for coherent detection, thereby consuming part of the received optical power budget; its effect on **coherent receiver sensitivity** depends on the loss location within the receive chain and the specific receiver configuration. In typical coherent systems employing forward error correction (FEC) with high coding gain, such a loss level remains acceptable for most practical link scenarios, and pre-amplification at the coherent receiver can compensate for it when additional margin is required.

The additional loss is likely related to laser-induced material modification, including a-Si formation, defect generation, and scattering arising from spatial nonuniformity of the modified region; the uniformity is in turn affected by the laser-processing parameters, the modification geometry, and the Gaussian intensity distribution of the femtosecond laser beam. Accordingly, further optimization of these factors, together with beam-shaping techniques such as flat-top illumination, is expected to improve modification uniformity and thus reduce the trimming-induced loss, thereby strengthening the practical viability of this post-fabrication trimming approach in silicon photonic coherent receiver applications.

References

[S1] Johnson, S. G., Povinelli, M. L., Soljačić, M., Karalis, A., Jacobs, S., Joannopoulos, J. D. Perturbation theory for Maxwell’s equations with shifting material boundaries. Phys. Rev. E, 65: 066611 (2002).

[S2] Snyder, A. W., Love, J. D. Optical Waveguide Theory. Chapman and Hall, 1983.

[S3] Garcia-Lechuga, M., Casquero, N., Siegel, J., Solis, J., Clady, R., Wang, A., Utéza, O., Grojo, D. Amorphization and ablation of crystalline silicon using ultrafast lasers: dependencies on the pulse duration and irradiation wavelength. Laser Photon. Rev., 18: 2301327 (2024).

[S4] Jellison, G. E. Jr., Modine, F. A. Parameterization of the optical functions of amorphous materials in the interband region. Appl. Phys. Lett., 69: 371–373 (1996).

[S5] Du, X., Zhou, L., Xu, W., Zhao, Y., Chen, M., Zhang, B., Chen, W. Modifying single-crystal silicon and trimming silicon microring devices by femtosecond-laser irradiation. Opt. Express, 32: 16199–16211 (2024).

[S6] Bonse, J. All-optical characterization of single femtosecond laser-pulse-induced amorphization in silicon. Appl. Phys. A, 84: 63–66 (2006).

[S7] Wang, Y., Zhang, Y., Li, Z., et al. Deep Silicon Amorphization Induced by Femtosecond Laser Pulses up to the Mid-Infrared. Adv. Opt. Mater., 9(14): 2100400 (2021).

[S8] de Dood, M. J. A., Polman, A., Zijlstra, T., van der Drift, E. W. J. M., Sinke, W. C. Amorphous silicon waveguides for microphotonics. J. Appl. Phys., 92: 649–653 (2002).
